# Book Reviews

**Published:** 1855-12

**Authors:** 


*Introductory Address delivered at the College of Physicians and Surgeons, New York, Oct. 16th,* 1855. By JOHN C. DALTON, M. D., Professor of Physiology and Microscopic Anatomy.

We would be sorry to promise a notice to *all* the introductories sent us, but this by our friend is one of such point and common sense that we cannot withhold our word of approbation. To describe its qualities negatively, it has no "big fat words," no *soaring,* no nonsense. Positively, it possesses acumen, neatness of style, freshness of thought, and logical accuracy. These are qualities belonging by birthright to the author, who has already acquired an enviable distinction as the foremost teacher of physiology in this country.

*The Anatomical Remembrancer; or, Complete Pocket Anatomist: containing a Concise Description of the Structure of the Human Body.* Second American, from the Fourth London Edition. With corrections and additions by C. E. Isaacs, M. D., Demonstrator of Anatomy in the University of New York. New York: S. S. & W. Wood. 1855.

The plan of this little manual seemed to us so faulty that we were inclined to condemn it at first sight. It is surprising, however, to notice the compactness of the descriptions. A single sentence is made to do double service, every word has a meaning and a place. No person could learn anatomy from it, but it would be really useful as a remembrancer for speedy reference. This is all it claims to be.

---

*How to Nurse Sick Children:* intended especially as a help to the Nurses at the Hospital for Sick Children; but containing directions which may be found of service to all who have charge of the young. New York: S. S. & W. Wood. 1855.

It was no itching for authorship that dictated this publication; a neat little manual not too long to be read, which teaches not how to physic and doctor, but how to *nurse* sick children. It is just such a little book as the physician might place in the hands of a young mother with a certainty of its usefulness. We commend it to the kind offices of the profession, not so much for themselves as for their patients.

For sale by Phinney & Co.

